# Therapy Efficacy of Idiopathic Ventricular Extrasystoles: A Real Life Study

**DOI:** 10.1155/2023/5590422

**Published:** 2023-10-27

**Authors:** Aliisa Lönnrot, Jaakko Inkovaara, Olli Arola, Tero Penttilä, Heikki Mäkynen, Katriina Aalto-Setälä, Sinikka Yli-Mäyry

**Affiliations:** ^1^Faculty of Medicine and Health Technology, Tampere University, Arvo Ylpön Katu 34, 33540 Tampere, Finland; ^2^Heart Hospital, Tampere University Hospital, Ensitie 4, 33520 Tampere, Finland

## Abstract

**Background:**

Ventricular extrasystoles (VESs) are common and often harmless in a healthy heart, but they can significantly affect the quality of life. If changes in lifestyle and antiarrhythmic medication are not enough, invasive and often curative catheter ablation can be considered. Better understanding of the conformation of VESs with a 12-lead ECG, as well as their precise localization, have increased their treatment with catheter ablation. Our goal was to determine whether the anatomical site of VES had an effect on procedure success. We also analyzed the safety of the procedure and patient-related factors affecting the results.

**Materials and Methods:**

In this retrospective study, we analyzed the medical records of 63 consecutive patients with multiple idiopathic VESs treated by catheter ablation at Heart Hospital, Tampere University Hospital, during 2017 and 2018. Patients with structural heart disease were excluded. Ablation success was estimated with two endpoints, primary and follow-up success.

**Results:**

The majority of the patients received treatment on the right ventricular outflow tract (66.7%), others on the left ventricle (17.5%), or the aortic cusp (9.5%). The site of origin remained unknown in four procedures (6.3% of patients). Primary success was observed in 48 procedures (76.2%). During the follow-up period of three months, the procedure was successful in 70.3% of the cases. The anatomical site of VES had no significant effect on either primary or follow-up success. Those with a successful follow-up result had a lower body mass index (BMI = 26.4) than those who had an unsuccessful result (BMI = 28.7; *p*=0.069); this did not reach statistical significance, potentially due to the small study population size. Complications were observed in three patients (4.5%). All of them were related to the catheter insertion site.

**Conclusions:**

For a symptomatic patient, catheter ablation is an effective and often fully curative treatment. The success rate was similar regardless of the site of VESs. This suggests that catheter ablation should also be assessed early on for other cases besides classic right ventricular outflow tract VESs. A high BMI was the only factor associated with a poor procedure success rate. The procedure itself is safe, and adverse effects are rare. The radiation dose is also low partly due to the current magnetic navigation method.

## 1. Introduction

Ventricular extrasystoles (VESs) are often harmless, and most patients are asymptomatic, or the symptoms are mild. However, VESs can worsen the quality of life by causing dizziness, palpitations, fatigue, a deterioration of physical performance, and syncopal attacks [1]. When the patient is experiencing symptoms, further clinical examinations are needed because VESs can be a sign of heart disease, such as coronary artery disease, hypertension, cardiomyopathy, or a congenital defect of the heart [2]. Frequent VESs can also produce a reversible form of left ventricular dysfunction and heart failure [3].

VES is the premature depolarization of the heart, originating at the ventricular level. Ventricular extrasystoles are found in 0.7–1% of clinically normal individuals when detected by a standard ECG and in 50% when detected by a 24-hour ambulatory (Holter) ECG. Prevalence increases with age [1, 3].

The goal for clinical examinations and the interpretation of ECG is to determine the presence, number, and origin of VESs and to assess whether they are benign or dangerous. Structural heart disease and ECG findings suggesting genetic disorders, such as long or short QT syndrome or Brugada syndrome, should be excluded before classifying VESs as harmless. In addition, unconsciousness events in the patient's history also require further diagnostic tests. Usually, VESs in a structurally normal heart, with no effect on hemodynamics, are benign. In addition, idiopathic harmless VESs should also stop at higher heart rates during exercise [2, 4, 5].

Twenty-four-hour ambulatory monitoring (Holter) reveals the number of VESs as well as their configuration, the time of day when they are mostly present, whether they originate from one or multiple sites, and also whether they are associated with the patient's symptoms. Exercise stress testing demonstrates whether physical activity increases or decreases the number of VESs. If the amount increases or if ischemia findings are present during stress testing, further examinations are required [6]. Asymptomatic patients should also undergo echocardiography to rule out structural or functional problems requiring more specific treatments. The need for magnetic resonance imaging (MRI) should be considered individually [6]. Some form of cardiac imaging (CT, coronary angiography, and MRI) is often important and performed on almost all patients with ventricular tachycardia. The primary aim is to treat the underlying heart disease, and only if this has been ruled out, ventricular extrasystoles are classified as idiopathic [2].

In this study, we assessed whether different sites of origin for VESs affect the success of a catheter ablation procedure. We also studied the safety of the procedure, focusing on adverse events and radiation time. In addition, we analyzed whether there were any patient-related factors which could affect the ablation success rate.

## 2. Materials and Methods

Our retrospective study included 63 consecutive patients who underwent a catheter ablation procedure for idiopathic VES in 2017 and 2018. The study was approved by Pirkanmaa Hospital District Research Services and fully complies with the Declaration of Helsinki. Because of the nature of this study, no informed patient consent was required or obtained. The average age was 52.8 years, and 50.8% of the patients were women. Other clinical features are presented in Table 1. All patients were asked about possible lifestyle factors that potentially increased the number of VESs—for example, sleeping habits, current stress, caffeine and alcohol consumption, the use of allergy medication, or other diseases. Other factors possibly affecting VESs, such as hypokalemia, anemia, and thyroid dysfunction, were excluded with laboratory testing. Factors generally known to cause VESs, such as cardiomyopathies and ischemic heart diseases, were examined and excluded, and all cases included in the study were classified as idiopathic. Echocardiography and a 12-lead ECG were performed on all patients before the ablation procedure. Other examinations are listed in Table 2.

Four of the patients underwent the same procedure twice during the study period, yielding a total of 67 procedures. When analyzing the site of origin and its effect on procedure success, we included only one procedure per patient (63 overall), selecting the first procedure for the four patients with repeated procedures. When analyzing only procedure-related facts, such as procedure time, adverse effects, and radiation time, we included all 67 procedures.

### 2.1. ECG

**Figure 1:**
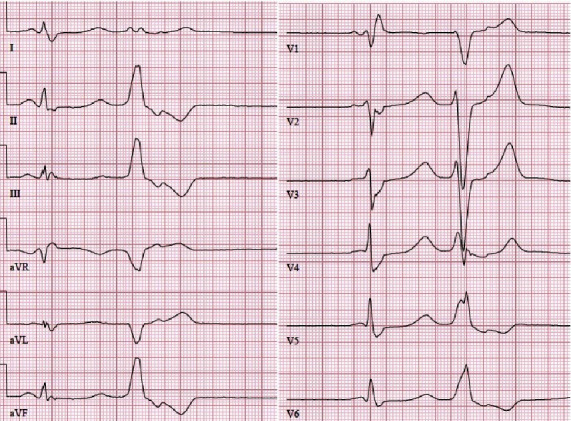
Ventricular extrasystole in a 12-lead ECG, originating from the right ventricular outflow tract (RVOT). The patient was a 62-year-old man with severe symptoms and over 42,000 ventricular extrasystoles during 24-hour Holter monitoring, who was ablated successful.

**Figure 2:**
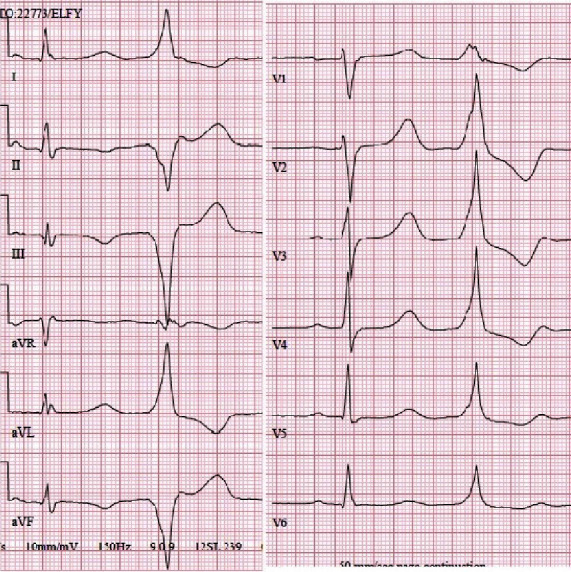
Ventricular extrasystole in a 12-lead ECG, originating from the left ventricle (LV). The patient was a 55-year-old woman with over 29,000 extrasystoles during 24-hour Holter monitoring, as well as palpitations, and reduced left ventricular function in echocardiography, and she was ablated successfully. At the follow-up, all symptoms had disappeared, only 200 extrasystoles were observed in Holter monitoring, and left ventricular function normalized.

**Figure 3:**
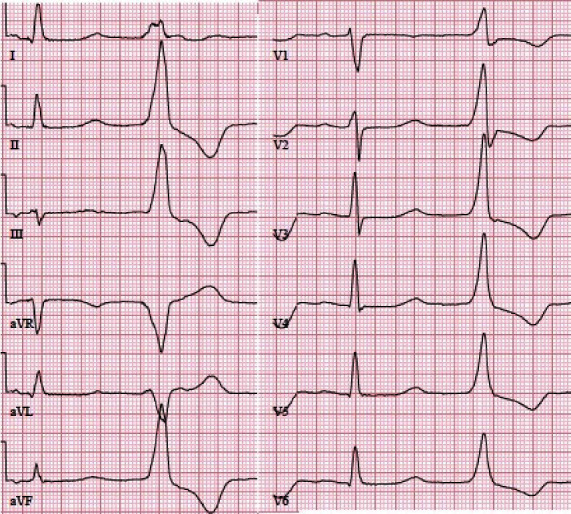
Ventricular extrasystole originating from the aortomitral continuity (AMC) area. The patient was a 67-year-old woman with very severe symptoms despite a rather low amount of extrasystoles (circa 5,000 in Holter monitoring). The extrasystole was localized to the AMC area and was ablated successfully.

**Figure 4:**
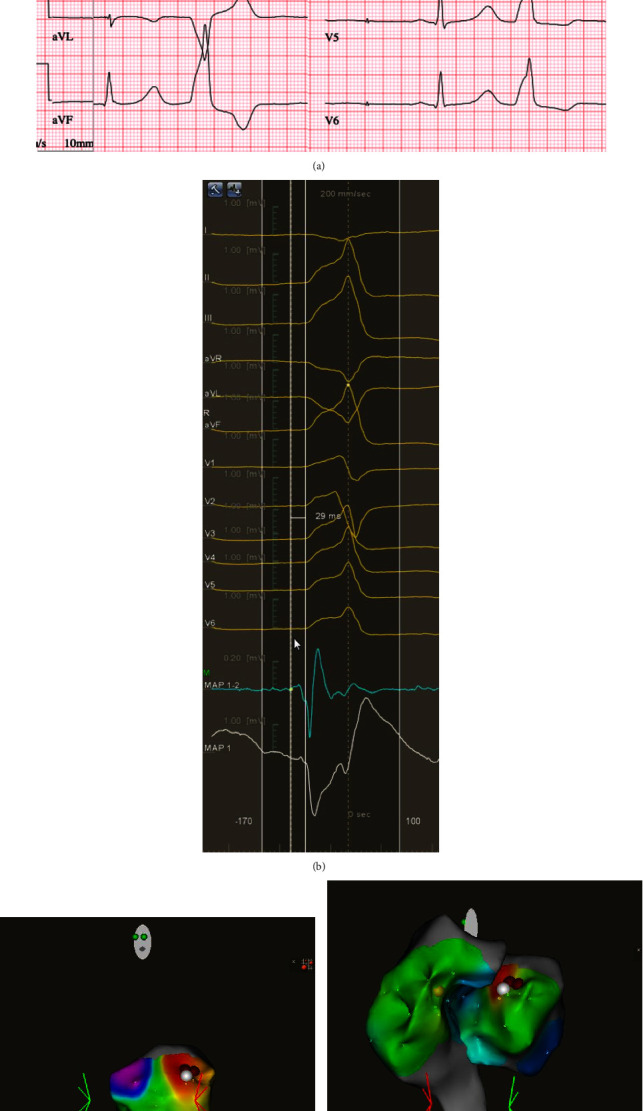
(a) Ventricular extrasystole in a 12-lead ECG, originating from the left coronary cusp (LCC). The patient was a woman aged 24 years with normal left ventricular function and 47,000 extrasystoles in Holter monitoring, experiencing palpitations and presyncope. (b) CARTO mapping shows an optimal intracardiac signal located ideally, 29 ms before the QRS complex, and comparable with a 12-lead ECG extrasystole. (c) Aortic valve mapping reveals the optimal site of the extrasystole located in the left coronary cusp (LCC) in the left anterior obtuse view (LAO). (d) Aortic root and right ventricular outflow tract (RVOT) mapping. Red dots indicate the successful ablation site in the left coronary cusp (LCC).

In all patients VES could be captured in a 12-lead ECG (see examples in Figures 1–4(a)). All VESs were monomorphic with fixed coupling intervals. ECGs were interpreted based on known ECG criteria for different sites of origin, such as bundle branch block configuration and precordial transition. The main criteria typical of different locations are summarized in Table 3. VES locating on the right side is treated if the patient prefers, but ablation of VES locating on the left side is considered only after antiarrhythmic medications are considered to be ineffective or not tolerated [7].

### 2.2. Catheter Ablation

In each case, a 3D electroanatomic map of the cardiac structures was constructed using CARTO (Biosense Webster, Inc.) in all ablation procedures (Figures 4(b)–4(d)). Detailed mapping was undertaken of the aortic valvular sinuses, the LV outflow tract, the basal LV septum, and the septal RV/RV outflow tract. Ablation was performed at the earliest site of activation and pace-map match using template matching software. The mapping and ablation of VESs were performed using the magnetic navigation system (Stereotaxis, Inc., St Louis, MO, USA) in conjunction with a cardiodrive motor unit. A soft magnetic catheter (Celcius RMT, 4 mm solid tip, Biosense Webster, Inc.) was navigated to the site of VES origin using a sequence of preinstalled magnetic vectors [10]. The origin of VESs was treated for 60 seconds with 35–40 W. Magnetic navigation and radiofrequency ablation were used in all but one of the catheter ablations [11].

With one patient, ablation was performed with the cryo method because of the patient's spinal cord stimulator. The procedure was unsuccessful. The spinal cord stimulator was later removed because the patient had extremely difficult symptoms, such as fatigue, shortness of breath, chest pains, and bradycardia of approximately 35 BPM. Approximately, 17% of the beats were VESs during Holter recording. The patient also had several visits to an emergency unit because of the symptoms, and at least once, the patient was hospitalized and monitored because of the symptoms. Removing the spinal cord stimulator enabled the use of magnetic navigation and RF ablation, and it was possible to treat VESs.

The site of origin was categorized into two groups: the right ventricular outflow tract and the left ventricle. The left side was then subcategorized into the aortic cusps (left and right coronary cusps) and other locations (Table 4).

Two endpoints were used to estimate the success of the procedure: primary success was judged at the end of the procedure, and follow-up success was evaluated three months after the procedure. Patients were monitored in the operation room for 30–40 minutes after ablation, and if VESs were not detected, the procedure was defined as primarily successful. At the three-month follow-up, the procedure was deemed successful if 500 or fewer VESs were seen in 24-hour Holter monitoring and the patient was asymptomatic. Some of these cases were further classified as partially successful if these criteria were not fully met, but the symptoms clearly eased and/or the number of VESs in a day significantly decreased. In unsuccessful procedures, there was no or little response to ablation, with the same subjective symptoms and number of VESs in Holter recording. It was possible that the procedure was primarily successful but not successful at the follow-up, and vice versa.

### 2.3. Statistical Analysis

Data were analyzed using SPSS Statistics version 26. Pearson's chi-squared test was used for categorical data. The independent samples *t*-test and the Mann–Whitney *U*-test were used for normally and nonnormally distributed data, respectively, to see if there were statistical differences between the two groups. We considered *p* values of 0.05 or lower to be statistically significant. The mean and standard deviation are presented for normal distributions and the median and quartiles for nonnormal distributions.

## 3. Results

The criteria for the ablation procedure were that the patient experienced symptoms and that the number of VESs was substantial. One of the patients was asymptomatic, but the decision was made to proceed with the ablation procedure because of the high number of VESs (approximately 40% of all beats in a day) which leads to increased risk for cardiomyopathy.

Sixty-three ablation procedures were taken into consideration when analyzing the success and ablation site. Forty-two ablation procedures (66.7%) were performed on the right ventricle area and 17 (27.0%) on the left side. In four procedures, the treatment site was unclear. The left ventricular sites as well as other anatomical sites are presented in Table 4. During the procedure, another arrhythmia mechanism was found in three patients. One had a concealed left-sided accessory pathway causing atrioventricular re-entrant tachycardia (AVRT), and two patients had a dual atrioventricular nodal pathway causing atrioventricular nodal re-entrant tachycardia (AVNRT). These were all treated successfully during the same procedure.

Partly due to the electroanatomical mapping and magnetic navigation methods used, the radiation dose was low and the radiation time was short. The radiation dose in radiofrequency ablation was 2.96 Gy·cm^2^ (dose area product; IQR 1.53−1.11), and the average radiation time was 4 minutes and 37 seconds (IQR 0 : 02 : 31−0:08 : 11). In cryoablation, both radiation parameters were higher. The variables related to the catheter ablation procedure are presented in Table 5, with values for cryoablation excluded.

Primary success was observed in 48 procedures (76.2%). We were able to obtain follow-up results for 35 patients (55.6%), among whom ablation was successful in 26 cases (74.2%). Eight of these procedures (total 22.9%) were partially successful. This meant that over 500 VESs were still observed in a 24-hour ECG recording after the ablation procedure, but the number was reduced when compared to the recording before ablation, and the symptoms clearly eased. Nine procedures (25.7%) were unsuccessful. The follow-up results of 28 patients (44.4%) were not available. Twenty-three of their procedures (82.1% of 26 cases) were primarily successful.

The anatomical site of VES had no significant effect on either primary or follow-up success. Primary success was determined in 81% (34 procedures) of right ventricle ablation, 90.9% (10 procedures) of left ventricle ablation, and 83.3% (5 procedures) of cusp ablation. There was no statistically significant difference between these groups (*p*=0.58). Follow-up success was accomplished in 75% (21 procedures) of the right ventricular procedures, in 80.0% (4 procedures) of the left ventricular procedures, and in 75% (3 procedures) of the cusp procedures of those whose follow-up results were available. Follow-up results were not significantly different between these groups (*p*=1.0).

Of the patients with a successful follow-up result, 92.3% also had a primarily successful ablation procedure. In contrast, only 22.2% of those whose follow-up result was not successful had a primarily successful procedure. Primary success predicted a successful follow-up result which was statistically significant (*p*=0.001). The median BMI of those who had a successful procedure was 26.4 (SD: 23.6–28.2), while the median BMI of those who had an unsuccessful procedure was 28.7 (SD: 26.2–34.7). However, the calculated *p* value remained too high to be considered statistically significant (*p*=0.069).

In 67 procedures completed, three adverse events (4.5% of the procedures) were reported. These occurred in three patients and were all related to the catheter insertion site. Two of the adverse events (a femoral artery pseudoaneurysm and a femoral arteriovenous fistula) required surgical intervention. The third was a significant hematoma and swelling of the catheter insertion site, which healed without any treatments.

## 4. Discussion

This study aimed to evaluate the feasibility of invasive catheter ablation as the treatment of benign, but quality of life-affecting ventricular extrasystoles (VESs), and to identify factors related to unsuccessful procedures. Medication is currently considered the first choice of treatment, although its effect on VES is only moderate. This study shows that if medical treatment is insufficient and the patient's symptoms are disturbing, catheter ablation is safe and effective regardless of the site of origin of the ventricular extrasystoles in real life.

Predisposing lifestyle factors known to cause idiopathic VES are caffeine, alcohol, stress, and insomnia [1, 12]. Many patients have disturbing subjective symptoms which decrease their quality of life. Related lifestyle adjustments can decrease symptoms. If lifestyle adjustments are not effective enough, medication is considered. However, the adverse and proarrhythmic effects of medication should always be considered. The effect of beta blockers on reducing ventricular arrhythmias is weak [6]. They may prolong the compensatory pause seen after the premature ventricular contraction, which the patient can experience as a heavy feeling, unpleasant pause, or dizziness. In our study, 90.5% of the patients had tried or actively used a beta blocker. It is likely that nearly 100%, if not all, had tried one at some point, and the medical records we used were lacking that information. Group I and III antiarrhythmic medication should only be started by a specialist. In our study, 15 patients (23.8%) were treated unsuccessfully with flecainide and three (4.8%) with amiodarone at some point. Patients using diuretics can sometimes benefit from potassium or magnesium supplements [13].

The catheter ablation results in our study are in line with those of previous studies. The majority of the patients (74.3% of those whose follow-up results were available) had a successful procedure. No randomized studies have been carried out on the topic, but the number of VESs has been found to decrease in approximately 74%–100% of patients after the procedure [6]. Studies have shown that the most common site of origin for VES is the right ventricular outflow tract [8]. Our study is in line with these results. However, the success rate in procedures performed on the left side and coronary cusps was comparable to procedures performed on the right side. The potential risk of adverse events has been reported to be slightly higher when the procedure is performed on the left side of the heart because a transseptal puncture is made. Adverse effects are, however, rare but potentially lethal, such as cardiac tamponade, thromboembolic complications, or aortic root puncture [14]. In our study, a transseptal puncture was performed in 18 procedures, and no adverse events occurred.

When planning the catheter ablation procedure, the number of VESs should not be the only factor guiding decision-making, but experienced symptoms and the patient's suitability for catheter ablation should also be taken into consideration. In the current study, the site of origin in one asymptomatic and severely obese patient was suspected to be on the left side after unsuccessful right-side mapping. The risks of transseptal puncture were estimated to be higher than that of the possible effects of VES on the patient's heart, and conservative treatment was thus chosen.

There can be various reasons behind the unsuccessful catheter ablation result. Male sex and older age have been linked to an unsuccessful result [15]. Also, one main reason is the site of origin–if it is located intramurally or epicardially, endocardial treatment may not be enough to burn the exact site [16]. Other reasons for failing catheter ablation include an error in localization and inability to position the ablation catheter at the target site [17]. RVOT as a site of origin has also been linked to a better success rate [18], but in our study, this location was not associated with a better outcome.

In some unsuccessful ablation, the site of origin was suspected to be intramural or epicardial during the procedure. This was based on the fact that, with thorough electroanatomic mapping of the endocardium, a near-perfect correlation was found, but endocardial site ablation did not terminate the VES. We are currently lacking ECG criteria based on which these VESs could have been reliably classified as epicardial or intramural, and epicardial ablation techniques are not commonly used. The ECG criteria for epicardial and intramural VES have been difficult to determine because successful ablation does not necessarily indicate the exact site of origin. Epicardially located VES can sometimes be eliminated from the endocardial side, if the formed lesion is deep enough and penetrates through the myocardium. Intramural VES can be eliminated in the same way [9].

Because VES is often a benign phenomenon, as was also the case in the present study, the safety of catheter ablation must be determined. When using modern magnetic navigation technology, the average radiation dose was low (median 3.3). Converted to mSv, the median dose per procedure is 0.33 mSv [19, 20]. For comparison, the radiation from thoracic X-ray imaging is approximately 0.1 mSv and yearly background radiation is roughly 6 mSv per person [21–23]. In our study, the median procedure time (2 hours and 27 minutes for all procedures) was similar compared to that in other studies [24, 25].

Adverse effects occurred in 4.5% of the procedures. They were all related to the catheter insertion site and may have been avoided by performing venous puncture under ultrasound control. We have used ultrasound control in every electrophysiological procedure since 2020. In our hospital, the right ventricle is often mapped first because transseptal puncture can increase the risk for complications [14]. In some cases, transseptal puncture can be avoided if the site of origin is found from the right side. If the ECG clearly indicates that the site of origin is most likely on the left side (LBBB configuration and early transition in leads *V*1-*V*2), the left ventricle can be mapped first.

No studies have previously been carried out on the effects of overweight on catheter ablation success of VESs and the permanence of the result. In our study, there was an indication of BMI affecting the results, but coincidence cannot be ruled out due to the small study population (*p*=0.069). This observation needs further studies and a larger study population to confirm the significance of BMI as the factor affecting procedure success. The connection between the recurrence of atrial fibrillation and overweight was observed in a meta-analysis of 51 studies, 16 of which indicated a connection between postablation atrial fibrillation and overweight. The risk of atrial fibrillation increased along with BMI [26]. The number of ventricular extrasystoles and their occurrence during physical stress has also previously been linked to overweight [27, 28].

The patients in our study had visited the cardiology unit or emergency department approximately two times because of the VES. The highest number of visits was nine. We were able to count these visits in 36 patients' medical records. Thirteen of them had visited the emergency department because of VES—one of them as many as four times. Catheter ablation of VES can reduce the number of visits in health care and save resources. A better understanding of VES and increasing awareness of catheter ablation as a possible treatment can enhance the steering of patients to specialized health care from primary health care. When catheter ablation techniques evolve, catheter ablation might even become the first-line treatment for symptomatic patients.

### 4.1. Limitations

The retrospective nature of the study protocol constitutes a limitation of this study. On the other hand, the study represents a real life aspect of catheter ablation treatment and, therefore, offers valuable information. Another limitation was the missing of follow-up results from a large proportion of patients (44.4%). Because of the significant connection between primary and follow-up success, however, it can be assumed that most of them benefited from the procedure.

## 5. Conclusions

The most important message of this retrospective study is the increased knowledge about the safety and suitability of VES for catheter ablation. With severely symptomatic patients, the treatment plan and suitability for catheter ablation should be assessed early on. The presented results of catheter ablation are in line with those of previous studies.

In our study, the site of origin did not affect the catheter ablation results, leading to the conclusion that the procedure should also be considered as an option in other cases besides classic RVOT ventricular extrasystoles. Catheter ablation is a safe procedure, and the radiation dose is mostly low. There were only a few adverse events, and all of them were related to the catheter insertion site. BMI affecting the result is an interesting aspect that needs further study. Steering symptomatic patients early on to a procedure that is fully curative in the best-case scenario saves resources by, for example, decreasing visits to the emergency department and is cost effective. In the near future, increasing use of digital methods will improve the long-term follow-up.

## Figures and Tables

**Table 1:** Patient characteristics and experienced symptoms categorized by follow-up success.

	Successful		Unsuccessful		*p* value
	*n* = 26		*n* = 9		
	*n*/mean/median	%/sd/*Q*1–*Q*3	*n*/mean/median	%/sd/*Q*1–*Q*3	
Female	13	50.0	5	55.6	
Age	49.5	17.1	56.5	9.9	0.369
BMI	26.4	23.6–28.2	28.7	26.2–34.7	0.069
Hypertension	7	26.9	3	33.3	—
Coronary artery disease	3	11.5%	1	11.1	—
DM2	0	0.0%	1	11.1	—
LVEF	60.0	57.3–65.8	60.0	55.0–66.5	0.940
LVEDD	52.0	49.0–59.0	55.0	52.0–61.0	0.244
Palpitations	25	96.2	8	88.9	—
Deteriorated physical health	12	46.2	5	55.6	—
Dizziness/presyncope	18	69.2	4	44.4	—
Syncope	2	7.7	1	11.1	—
QT time	407	38.6	434.0	26.3	0.610
QTc time	423.6	39.0 (max. 515)	448.8	33.0 (max. 500)	0.930
Number of VES/24 h before ablation	17,484.0	6,429.0−27,696.0	20,322	5,687.0−38,146.0	—
Number of VES/24 h after ablation	135.0	13.0−1,366.0	16,529	8,162.0−34,534.0	—
Primarily successful ablation	24	(92.3%)	2	(22.2%)	0.001

BMI, body mass index; DM, diabetes mellitus; EF, ejection fraction; LV, left ventricle; LVEDD, left ventricular end-diastolic diameter; VES, ventricular extrasystole.

**Table 2:** Examinations performed on patients before the catheter ablation procedure.

Clinical examination	*n* = 63 patients
12-lead ECG	63 (100%)
Cardiac ultrasound imaging	63 (100%)
Cardiac MRI	27 (42.9%)
Stress exercise test	45 (71.4%)
Hospital visits before procedure	2.5 (range 1–9 visits)
Elective patients	27 (42.9%)

**Table 3:** Interpretation of ECG [8, 9].

Site of origin	BBB	Precordial transition	Lead I	Lead *V*1	Other specific criteria
RVOT	LBBB	*V*3–*V*6	Missing S waves	RS or QS	
Anterior wall	LBBB	*V*4–*V*6	Dominant R waves	Deeper S waves	Wider QRS > 140 ms, notching
Septum	LBBB	*V*3-*V*4	QS	Smaller S waves	
LVOT	RBBB or atypical LBBB	*V*1–*V*3	S waves can be present		
AMC	RBBB, rarely LBBB	*V*2-*V*3	rS	qR	RS pattern in lead *V*2
LCC	RBBB	*V*1-*V*2			R wave amplitude lead III/lead II > 0.9
RCC	LBBB	*V*2-*V*3	QS		R wave amplitude lead III/lead II < 0.9

BBB, bundle branch block; RVOT, right ventricular outflow tract; LVOT, left ventricular outflow tract; AMC, aortomitral continuity; LCC, left coronary cusp; RCC, right coronary cusp.

**Table 4:** Site of origin of VES based on the catheter ablation procedure.

Site of origin	Patients *n* = 63	Primary success rate^*∗*^*n* = 63	Follow-up success rate^*∗*^*n* = 35
RVOT	42 (66.7%)	34 (81.0%)	21 (75.0%)
LV	11 (17.5%)	10 (90.9%)	4 (80.0%)
LVOT	2	1	1
AMC	3	3 (100%)	—
Anterolateral papillary muscle	1	1	0
Septum	1	1	1
Sinus coronarius	1	0	—
Ablation line to the left posterior fascicle	1	1	1
Septum, near mitral annulus	1	1	1
Septum, posteriorly near mitral annulus	1	1	—
Coronary cusps	6 (9.5%)	5 (83.3%)	3 (75%)
LCC	4	3	2
RCC	2	2	1
Site of origin remained unclear	4 (6.3%)	0%	0%

RVOT, right ventricular outflow tract; LV, left ventricle; LVOT, left ventricular outflow tract; AMC, aortomitral continuity; LCC, left coronary cusp; RCC, right coronary cusp. ^*∗*^When comparing the primary success rate and the follow-up success rate between different sites of origin groups, the *p* values were 0.58 and 1.0, respectively. The primary success rate was obtained from all 63 patients. The follow-up success rate was obtained from 35 patients.

**Table 5:** Variables related to the catheter ablation procedure.

Variables during ablation procedures	*n* = 67
Procedure time (hh : mm : ss)	2 : 27 : 00 (1 : 52 : 00, 3 : 08 : 00)
Cryoablation excluded (*n* = 65)	2 : 27 : 00 (1 : 52 : 00, 3 : 08 : 00)
Cryoablation (*n* = 2)	3 : 20 : 30 (ranging from 2 : 13 : 00 to 4 : 27 : 00)
Radiation time (hh : mm : ss)	0 : 05 : 11 (0 : 02 : 31, 0 : 08 : 11)
Cryoablation excluded (*n* = 65)	0 : 04 : 35 (0 : 02 : 25.50, 0 : 08 : 05.50)
Cryoablation (*n* = 2)	0 : 45 : 10 (ranging from 0 : 20 : 19 to 0 : 45 : 10)
Radiation dose (Gy·cm^2^)	3.3 (1.53, 11.1)
Cryoablations excluded (*n* = 65)	2.96 (1.53, 11.1)
Cryoablation (*n* = 2)	61.76 (ranging from 31.72 to 91.8)
Radiation dose (mSv)	0.33 (0.153, 1.11)
Adverse events	3 (4.5%)
Femoral artery pseudoaneurysm	1
Femoral arteriovenous fistula	1
Significant hematoma and swelling of the catheter insertion site	1

Number of procedures = 67.

## Data Availability

The data used to support the findings of this study are available from the corresponding author upon request.
